# Liquid chromatography–tandem mass spectrometry for the simultaneous quantitation of ceftriaxone, metronidazole and hydroxymetronidazole in plasma from seriously ill, severely malnourished children

**DOI:** 10.12688/wellcomeopenres.11728.2

**Published:** 2018-01-30

**Authors:** Martin Ongas, Joseph Standing, Bernhards Ogutu, Joseph Waichungo, James A. Berkley, Karin Kipper

**Affiliations:** 1 Center for Research in Therapeutic Sciences, Strathmore University, Ole Sangale Road, Nairobi, Kenya; 2 KEMRI-Centre for Clinical Research, Nairobi, Kenya; 3 Inflammation, Infection and Rheumatology Section, UCL Great Ormond Street Institute of Child Health, 30 Guilford Street, London, WC1N 1EH, UK; 4 Paediatric Infectious Diseases Research Group, Institute for Infection and Immunity, St. George's, University of London, Cranmer Terrace, London, SW17 0RE, UK; 5 KEMRI-Wellcome Trust Research Programme, Kilifi, Kenya; 6 Centre for Tropical Medicine & Global Health, Nuffield Department of Medicine, University of Oxford, Oxford, UK; 7 The Childhood Acute Illness & Nutrition (CHAIN) Network, Lenana Place, Nairobi, Kenya; 8 Analytical Services International, St George’s University of London, Cranmer Terrace, London, SW17 0RE, UK; 9 Institute of Chemistry, University of Tartu, Tartu, Estonia

**Keywords:** LC-MS/MS, ceftriaxone, metronidazole, complicated severe acute malnutrition, ultrafiltration, protein binding

## Abstract

We have developed and validated a novel, sensitive, selective and reproducible reversed-phase high-performance liquid chromatography method coupled with electrospray ionization mass spectrometry (HPLC–ESI-MS/MS) for the simultaneous quantitation of ceftriaxone (CEF), metronidazole (MET) and hydroxymetronidazole (MET-OH) from only 50 µL of human plasma, and unbound CEF from 25 µL plasma ultra-filtrate to evaluate the effect of protein binding. Cefuroxime axetil (CEFU) was used as an internal standard (IS). The analytes were extracted by a protein precipitation procedure with acetonitrile and separated on a reversed-phase Polaris 5 C18-Analytical column using a mobile phase composed of acetonitrile containing 0.1% (v/v) formic acid and 10 mM aqueous ammonium formate pH 2.5, delivered at a flow-rate of 300 µL/min. Multiple reaction monitoring was performed in the positive ion mode using the transitions *m/z*555.1→ *m/z*396.0 (CEF), *m/z*172.2→ *m/z* 128.2 (MET), *m/z*188.0→ *m/z*125.9 (MET-OH) and *m/z*528.1→ *m/z* 364.0 (CEFU) to quantify the drugs. Calibration curves in spiked plasma and ultra-filtrate were linear ( *r ^2 ^*≥ 0.9948) from 0.4–300 µg/mL for CEF, 0.05–50 µg/mL for MET and 0.02 – 30 µg/mL for MET-OH. The intra- and inter- assay precisions were less than 9% and the mean extraction recoveries were 94.0% (CEF), 98.2% (MET), 99.6% (MET-OH) and 104.6% (CEF in ultra-filtrate); the recoveries for the IS were 93.8% (in plasma) and 97.6% (in ultra-filtrate). The validated method was successfully applied to a pharmacokinetic study of CEF, MET and MET-OH in hospitalized children with complicated severe acute malnutrition following an oral administration of MET and intravenous administration of CEF over the course of 72 hours.

## Introduction

Serious infections are common in children, especially those with severe acute malnutrition (SAM) admitted sick to hospitals, with over 50% of patients estimated to be infected at any one time ^1, 2^. Mortality remains high in this patient group, despite implementation of current treatment guidelines ^3^. Although empiric antibiotics are routinely given ^4– 7^, it is not clear whether the currently recommended regimen is the most effective in the context of increasing antimicrobial resistance (AMR), and moreover whether expected therapeutic levels are achieved in this group of patients.

**Figure 1.**
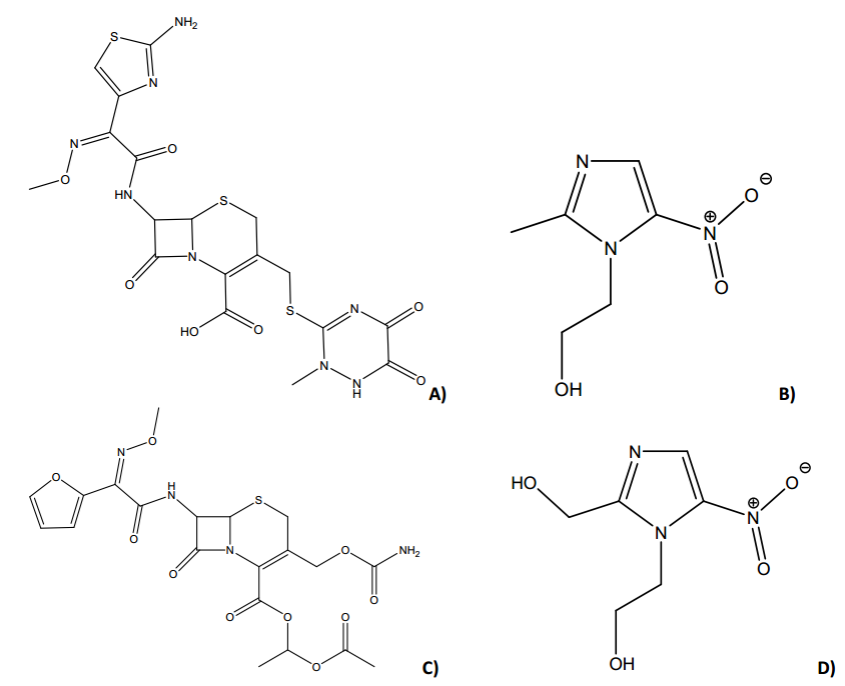
Chemical structures of ceftriaxone ( **A**), metronidazole ( **B**), hydroxymetronidazole ( **D**) and cefuroxime axetil, IS ( **C**).

To resolve this question, a large clinical trial of metronidazole (MET) and ceftriaxone (CEF) versus standard care (penicillin or ampicillin plus gentamicin) is planned. However, first, a study of the pharmacokinetics (PK) of MET and CEF is needed in order to optimize the dosing strategy in severely malnourished children, since they may have altered absorption, body composition, volume of distribution, available plasma proteins for binding, or metabolism and elimination through hepatic and renal pathways ^8, 9^. A quantitative determination of MET and CEF in plasma is essential in order to evaluate the pharmacokinetics of these co-administrated antibiotics ( Figure 1).

Previous studies have indicated the activity of MET and its two principle metabolites, 1-(2-hydroxyethyl)-2-hydroxymethyl-5-nitroimidazole (the "alcohol" metabolite, MET-OH) and 2-methyl-5-nitroimidazole-1-acetic acid (the "acid" metabolite) against a broad range of anaerobic bacteria ^10, 11^. In this study however, we focus on the major active metabolite (the “alcohol” metabolite). Several methods have been reported for quantification of either MET ^12– 15^ or MET and its metabolites ^11, 16^ in human plasma or serum. O’Keefe *et al*. ^11^ evaluated the activity of the metronidazole metabolites against anaerobic bacteria; however, the LC-UV method was limited in quantifying lower levels of the metabolites in a biological matrix due to its low sensitivity and poor selectivity. Silva *et al*. ^12^ developed an HPLC-MS-MS method for the quantitation of metronidazole in plasma. The method required large sample volumes and complex sample preparation steps, with large volumes of extraction solvents.

CEF, like other β-lactam antibiotics, is highly protein bound. Wong *et al*. ^17^ reported average protein binding of 89.5%. It has also been noted that ceftriaxone protein binding is nonlinear, becoming saturated at higher concentrations and linked with serum albumin concentrations in critically ill patients ^18^.

Given the significant effects of protein binding on clinical exposure to highly bound drugs ^17, 19– 23^, and given that the free drug is important for antimicrobial effect, it was necessary to develop a method to measure the unbound ceftriaxone appropriate for use in seriously ill malnourished children. Some of the methods reported previously ^24, 25^ give approaches to measurement of unbound fractions of compounds using equilibrium dialysis, which are more prone to environmental interference and much more laborious in sample preparations. Other methods involved the use of HPLC with UV detection, but did not consider the protein binding of CEF ^26– 29^.

We aimed to develop the first simultaneous HPLC-ESI-MS/MS method for rapid, simple, reliable, sensitive and selective quantitation of MET, CEF and MET-OH in a small volume (50 µL) of human plasma, and unbound CEF from (25 µL) plasma ultra-filtrate.

## Materials and methods

### Chemicals

Ceftriaxone sodium (CEF; batch no. 3.2, purity 90.4%; MW=554.58 g/mol), metronidazole (MET; batch no. 2.1, purity 100%, MW=171.15 g/mol) and cefuroxime axetil (CEFU, batch no. 4.0, purity 97.3%, MW=510.47 g/mol) were purchased from European Directorate for the Quality of Medicines and Healthcare (Strasbourg, France). Hydroxymetronidazole (MET-OH; Lot no. 4276, purity 98.2%, MW=187.15 g/mol) was purchased from LGC (Teddington, UK). Acetonitrile and methanol (both LC-MS grade), formic acid (85%; AnalaR ^®^grade) and ammonium formate (AnalaR ^®^grade) were purchased from Sigma Aldrich (St. Louis, MO, USA). Deionized water was prepared using a Smart2 Pure ^TM^ water purification system (Thermo-scientific, Niederelbert, Germany). Blank human plasma with Li-heparin for the preparation of calibrators and quality controls was obtained from Kenya Medical Research Institute, Centre for Clinical Research (Nairobi, Kenya). The matrix used to quantify free fraction of ceftriaxone was plasma ultrafiltrate obtained by ultrafiltration of drug-free plasma.

### Sample preparation

***Total drug.*** To a 50 µL aliquot of plasma (blank, standard, quality control, or patient sample) 200 µL of internal standard (CEFU; of a 1.25 µg/mL solution in acetonitrile) was added. The 1.5 mL polypropylene tubes were vortex-mixed for 3 minutes to precipitate the plasma proteins, followed by centrifugation (4000 x g; 10 min, 4°C). The supernatant (100 µL) was transferred into another clean 1.5 mL polypropylene tube and diluted with 400 µL of 20% methanol in water. The samples were vortex-mixed for 3 minutes and submitted for analysis by LC-MS/MS.

***Unbound ceftriaxone.*** About a 300 µL aliquot of patient plasma was taken into a clean 1.5 mL polypropylene tube and incubated on a Grant JB Series incubation bath (Grant Instruments, Cambridge, UK) at 37°C for 1 h, then transferred into Centrifree ^®^ Ultrafiltration Device (Merck Millipore Ltd, Darmstadt, Germany) and centrifuged on a Thermo Fisher Scientific SL 40R centrifuge (2000 x g; 30min, 37°C). 25 µL sample ultra-filtrate was taken into another clean 1.5 mL polypropylene tube; internal standard solution (200 µL, 1.0 µg/mL) in acetonitrile was added to the sample and diluted to 1 mL with 20% methanol in water. The samples were vortex-mixed for 3 min and submitted for analysis by LC-MS/MS. Calibrators and quality control (QC) samples were prepared by ultrafiltration of blank plasma after 1h incubation at 37°C, 200 µL aliquots the ultrafiltrate were spiked with 50 µL of CEF working solutions to produce 0.4, 12, 24, 48, 96, 150, 220, 300 µg/mL CEF and 1.2, 120, 240 µg/mL QCs.

### Preparation of analytical standards

Stock solutions of CEF (5 mg of the base/mL), MET and MET-OH (both 1 mg/mL) were prepared by dissolving an appropriate amount of each compound in 20% methanol. The stock solutions were further serially diluted with 20% methanol to make working standard solutions used to spike the blank plasma to produce 0.4, 12, 24, 48, 96,150,220, 300 µg/mL CEF and 1.2, 120, 240 µg/mL QCs; 0.05, 1.0, 2.0, 4.0, 8.0, 16.0, 32.0, 50 µg/mL MET and 0.15, 20, 40 µg/mL QCs; 0.02, 0.8, 1.6, 3.2, 6.4, 13, 20, 30 µg/mL MET-OH and 0.06, 12, 24 µg/mL QCs. Stock solution of CEFU (IS) was prepared by dissolving appropriate amount of the compound in acetonitrile, the stock solution was serially diluted with acetonitrile to make working standard solutions of 1.25 µg/mL and 1 µg/mL. All the stock solutions were stored at -20°C, protected from light (in amber sample vials) and used within three months.

### Chromatographic conditions

The equipment consisted of an Agilent Technologies HPLC-ESI-MS/MS system (Santa Clara, CA, USA), composed of a 1260 µ Quaternary Pumps, 1260 Autosampler and 1260 Thermosetting Column Compartment (TCC). Chromatographic separation was performed on a Polaris 5 C18-A (150 mm x 3.0 mm I.D; 3.0 µm particle size) analytical column from Agilent Technologies (Santa Clara, CA, USA) with a C18 guard cartridge (4 mm x 3.0 mm, 3.0 µm) (Phenomenex, Torrance, CA, USA) maintained at 30°C. The mobile phase consisted of (A) 10mM aqueous ammonium formate pH 2.5 and (B) 0.1% formic acid in acetonitrile. A linear gradient elution was used to deliver the mobile phase, 40% solvent B at time 0 min, and 100% from 1.8 min, to 5.5 min, and back to 40% from 6 min to 12 min, (re-equilibration step). The flow rate was set at 300 µL/min, an injection volume of 5 µL was used to optimize the drug signals and for analysis.

### Mass spectrometry

Mass spectrometric detection of analytes was performed on a 6410 Triple Quadrupole Mass Spectrometer with an Electrospray Ionization (ESI) source from Agilent Technologies (Santa Clara, CA, USA) in positive ionization mode. Nitrogen was used as the nebulizing, desolvation and collision gas, the optimized ion source parameters were: ion spray voltage 4.0 kV, exit potential 7V, RF lens 0.5 V.

Source temperature was 100°C and desolvation temperature 300°C. High purity nitrogen from Genius NM32LA generator (Peak Scientific, Scotland, UK) was used as both sheath and auxiliary gas set at 20 l/min and 12 l/min, respectively.

Multiple reaction monitoring (MRM) was employed for the data acquisition, the analytical parameters optimized for the compounds were declustering potentials (DP) and collision energies (CE) ( Table 1), and the scan dwell time was set at 500 ms. for each channel. Data acquisition and analysis were accomplished with Mass Hunter software (version A.02.00; Agilent Technologies).

**Table 1.** Compound optimization parameters for ceftriaxone (CEF), metronidazole (MET), hydroxymetronidazole (MET-OH) and cefuroxime axetil (CEFU), including multiple reaction monitoring (MRM) transitions, declustering potentials (DP) and collision energies (CE).

Compound	Precursor ion	MRM Transition (m/z)	DP (V)	CE (eV)
CEF	[M+H] ^+^	555.1→ 396.0	60	18
MET	[M+H] ^+^	172.2→ 128.2	100	15
MET-OH	[M+H] ^+^	188.0→ 125.9	100	15
CEFU	[M+NH _4_] ^+^	528.1→ 364.0	60	18

### Validation

Method validation was performed as per the US Food and Drug Administration Guidance for Industry Bioanalytical Method Validation ^30^. The method was validated for selectivity and sensitivity, inter-day and intra-day accuracy and precision, extraction recovery, matrix effect and stability. Method’s linear range was evaluated and lower limit of quantification was set to fit for purpose for the actual clinical trial samples. Carry-over was assessed in accordance with the European Medicines Agency guideline ^31^.

Selectivity of the method was assessed and assured by analysis of six blank plasma samples from different sources, each blank sample was tested for interference using the proposed extraction procedure and chromatographic/mass spectrometric conditions and compared with those obtained with an aqueous solution of the analyte at a concentration near to the lower limit of quantification (LLOQ). A plasma sample fortified with cefadroxil and cefaclor was also processed and analyzed.

ExtractionThe standard curves were obtained through analysis of calibration standard plasma and ultra-filtrate (for free CEF) samples and plotting of peak area ratio of MET, CEF and ME-OH versus the corresponding nominal concentrations. The linearity of the standard curves were evaluated using least-squares linear regression analysis.

The analytical extraction recovery was determined by comparing the response of extracted quality control plasma samples with the response of post extracted plasma samples spiked at similar concentrations to the quality control samples.

To evaluate the inter-assay precision and accuracy, six replicates of quality control plasma samples were analyzed together with one independent calibration standard curve, this was done in three consecutive days; while intra-assay precision and accuracy were evaluated through analysis of quality control plasma samples in replicate of six in the same day. Inter-assay and intra-assay precision were expressed as coefficient of variation (CV%).

The accuracy was expressed as the percent ratio between the experimental concentrations and the nominal concentration for each sample. A similar assessment was done for plasma ultra-filtrate to determine the accuracy and precision for the unbound ceftriaxone.

Stability (ST%) studies were evaluated via sample and solution concentrations, where:

$$ST\%=\frac{c_{t}}{c_{0}}\times \text{100\%}.\;(i)$$

ST% is the stability of the chemical compound in the sample over the period of time. c _*0*_ is the initial concentration, determined without introducing any extra pauses in the analysis process. c _*t*_ is the concentration obtained after the storage period with time *t*.

Sub-stock solution stability was evaluated for CEF, MET and MET-OH, by comparing the response generated from the same solution at preparation and after being stored at -20°C for a period of 28 days. All the analytes were found to be stable within the period investigated and fresh stock solutions were prepared thereafter, fresh IS solution was prepared daily from weighing during the method validation and study samples analysis. The stability was reported as coefficient of variation between the initial concentration and the concentration at day 28.

Spiked plasma samples were subjected to three freeze-thaw cycles at -20°C and the analytes concentrations assessed after the third cycle. This was done also for plasma ultra-filtrate spiked with CEF to assess the stability of free ceftriaxone in calibrators and quality control samples.

Bench-top stability was evaluated by keeping plasma samples at low and high quality control levels at ambient temperatures (< 28°C) for at least 8h then processed and analyzed. Selected ambient temperature covered the temperature range for study samples, as ambient temperatures remained < 28°C.

Processed sample stability was assessed by letting the samples stay in the autosampler at 18°C for 24h and then they were analyzed the following day. This was done to ensure data integrity in case of equipment failure and initiation of a re-run.

Long term stability of the analytes was studied over a period that covered the duration of storage of the study samples from collection to the last sample analysis, this ensured that the integrity of study samples was not compromised over the period of storage. To investigate long term stability, two sets of sample aliquots were prepared at concentrations corresponding to low and high quality control levels. The first set was processed and analyzed at day 1 and the second set after 90 days of storage at -20°C. The analyte concentrations in the plasma and ultra-filtrate samples at 90 days of storage was compared with those obtained on day 1 to determine the percentage stability.

To assess carry-over, a processed blank sample was injected after a high concentration calibration standard at the upper limit of quantification (ULOQ) and the peak response in blank sample determined.

Two different methods were used to access and determine matrix effect. In the first method, regions of ion suppression or enhancement were evaluated by direct post column infusion of a mixture of analytes and IS at high concentration at the rate of 10 μL/min, while injecting a blank extracted plasma. In the second method, matrix effect (ion enhancement) was evaluated for MET in six different lots of plasma by comparing the response of post extracted plasma samples spiked with 0.15 µg/mL (LLOQ) and 40 µg/mL (ULOQ) of metronidazole with the response of neat standard solutions spiked at similar concentrations.

Incurred sample reanalysis was done 90 days after the initial study sample analysis. A subset of subject samples (25 samples) were selected from randomly picked study participants and analyzed against freshly spiked calibrators and QCs. The percentage variation in the two analyses were determined by:

$$Variation\;\%\;=\;\frac{\left(Rc–Oc\right)}{Mc}\;\;\times \;100\;\;\;\;\;\;\;\;\;\;\;\;\;\;\;\;\;\;\;\;\;\;\;\;\;\;\;\;\;\;\;\;\;\;\;\;\;\;(ii)$$

Where: Variation% is the percentage difference between the initial analysis and the reanalysis concentrations, Rc is the repeat analysis concentration measured, Oc is the initial analysis concentration measured, Mc is the mean of the initial and repeat analysis concentrations.

## Results and discussion

### Method development and chromatographic separation of the analytes

Ceftriaxone is an acidic compound possessing a β-lactam ring in its structure ( Figure 1A). Like many β-lactam antibiotics, CEF is more susceptible to chemical and biological degradation due to its labile β-lactam ring ^32, 33^. Metronidazole on the other hand is slightly basic and fairly resistant to degradation ^34, 35^. This work is unique and novel, designed to develop a method that would be useful in simultaneous assay of CEF, MET and MET-OH from only 50 µL of human plasma, and unbound CEF from 25 µL plasma ultra-filtrate based on the physicochemical properties of these compounds and the area of method application. Moreover, the concerns raised by Berezhkovskiy *et al*. ^22^ on temperature dependency of protein binding and the need to maintain the physiological temperature (37°C) through the sample processing time were considered in sample pretreatment.

The method took into account the therapeutic and overdose concentration ranges. The method has been validated and proved to be reliable for the determination of the drugs in human plasma. During the method development, several chromatographic conditions were optimized for all analytes such as the mobile phase composition, pH and various flow rates. Various ratios (80:20, 70:30, 60:40 v/v) of acetonitrile and 10 mM ammonium formate were tested as starting eluent for chromatographic separation. The variation in the mobile phase led to considerable changes in the chromatographic parameters, like peak symmetry and retention time. The pH effect showed that optimized conditions are reached when the pH value of the buffer is adjusted to 2.5 with formic acid, producing well resolved and sharp peaks for all analytes assayed. Henceforth, in the present method the pH adjusted to 2.5 and the chosen LC gradient ensured sharp chromatographic peaks with the best possible baseline-resolved separations of CEF, MET, MET-OH and CEFU (IS) within 4 minutes with a total runtime of 12 minutes. With the optimized MRM transitions, the stable and most intense product ions of CEF ( *m/z* 396.0), MET ( *m/z* 128.2), MET-OH ( *m/z* 125.9) and CEFU ( *m/z* 364.0) were detected ( Figure S1).

### Method validation

***Selectivity.*** All the lots of blank plasma used for selectivity studies met the acceptance criteria, no significant interferences at the retention times of the analytes or internal standard were found. Figure 2 shows the typical chromatograms of extracted blank plasma, blank plasma spiked with IS (Zero sample), a spiked plasma sample with the analytes at LLOQ and ULOQ level. It can be seen that there were no interfering peaks from endogenous compounds observed at the retention times of the analytes and the IS. Moreover, no interference was observed from plasma samples fortified with commonly used β-lactam antibiotics (cefadroxil and cefaclor), processed and analyzed as described under the proposed sample preparation procedure.

**Figure 2A.**
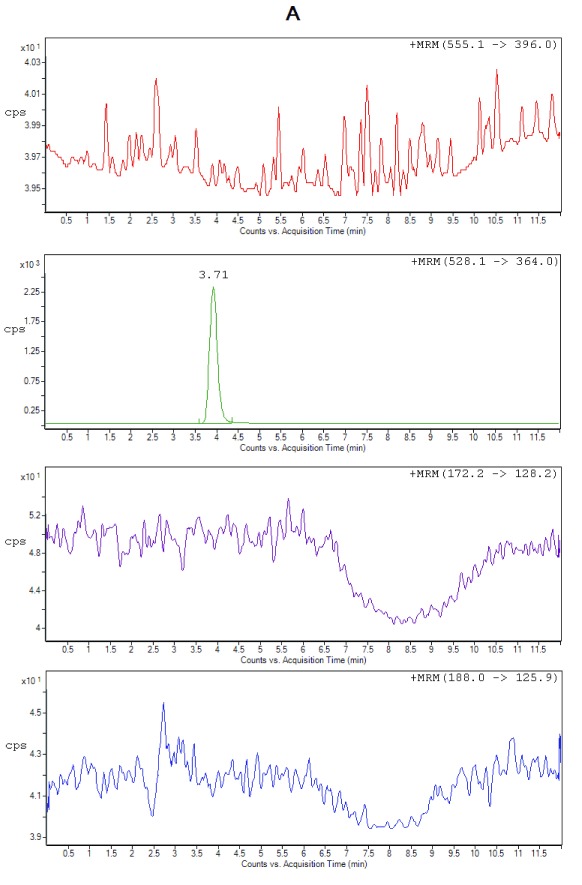
Representative chromatograms from extracted zero sample (with IS only), cefuroxime (RT 3.71 min).

**Figure 2B.**
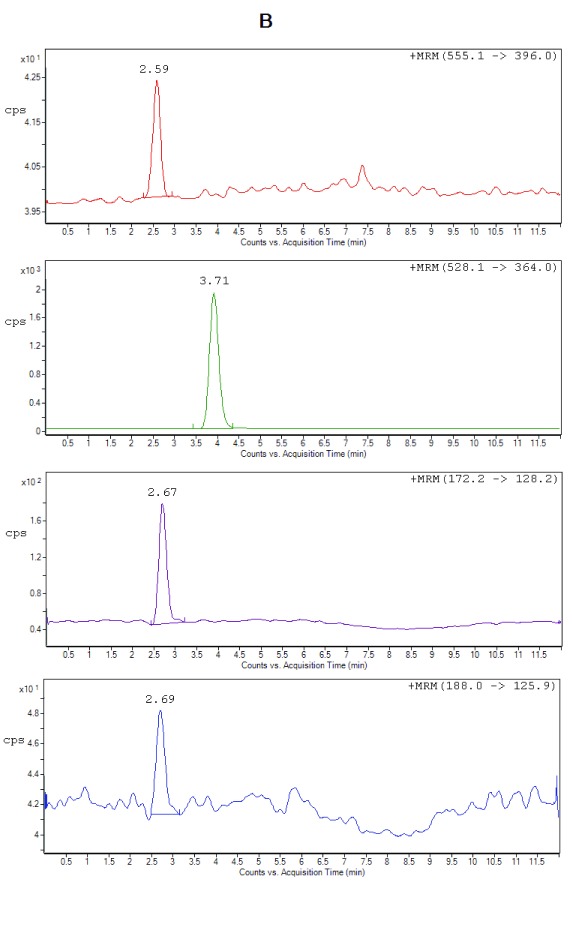
Representative chromatograms of ceftriaxone (RT 2.59 min), metronidazole (RT 2.67 min), hydroxymetronidazole (RT 2.69 min), and cefuroxime (IS) (RT 3.71 min) from extracted spiked plasma at LLOQ.

**Figure 2C.**
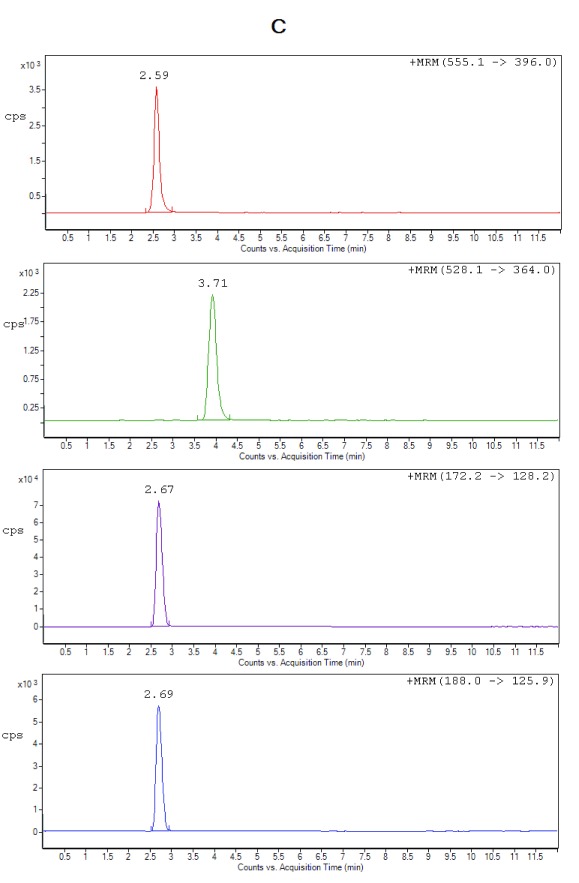
Representative chromatograms of ceftriaxone (RT 2.59 min), metronidazole (RT 2.67 min), hydroxymetronidazole (RT 2.69 min) and cefuroxime (IS) (RT 3.71 min) from extracted spiked plasma at ULOQ.

***Calibration curves and limit of quantification.*** Calibration curves were constructed by plotting peak area ratios of analytes and IS against the nominal concentrations of CEF, MET and MET-OH. The curves for drugs spiked in plasma were found to be linear over the concentration ranges of 0.4–300 µg/mL (CEF), 0.05–50 µg/mL (MET) and 0.02–30 µg/mL (MET-OH). A weighted (1/ *x* ^2^) linear regression model was used due to the wide range of concentrations covered by the calibration graphs. The choice of this regression model was based on all available data from the validation phase, in light of this the method proved to be reliable in terms of accuracy and reproducibility over the entire calibration range ( Table S1). The coefficients of variation of the slopes of six calibration curves were 9.8% (MET), 9.4% (CEF), 6.7% (MET-OH) and 7.2% (CEF in ultra-filtrate). The LLOQs for the method were set by the needs of the clinical trial. The LLOQ is the lowest standards on the calibration curve that the method is able to identify and whilst still providing discrete and reproducible results with a precision ≤ 20% and accuracy within 80%–120% ( Table 2). The limits of detection (LODs) were determined as the lowest concentration of the analyte at which the signal to noise (S/N) ratio exceeded 3:1 ^30^. ULOQ values were determined from anticipated peak concentrations ranges of the analytes, and ensuring that the calibration points met the accuracy and reproducibility criteria of method validation.

***Extraction recovery.*** Protein precipitation with acetonitrile was used to extract the analytes and the IS from plasma samples, this method was found to be efficient given the small sample volume (50 µL) used that would otherwise be impossible to use with the liquid-liquid extraction techniques employed in previously reported publications ^12, 13, 15^ for MET and ^26– 29^ for CEF. This still yielded higher recoveries with better reproducibility ( Table S2).

***Accuracy and precision.*** Accuracy of the method for the analytes in plasma were between 90.0%–105.5%, and precision, measured in CV%, was always lower than 8.5%, depicting the high precision of the method. The accuracy of the method for CEF in ultra-filtrate was between 93.6%–107.2% and a precision lower than 8.1% ( Table 2).

**Table 2.** Intra-assay and inter-assay accuracy and precision of metronidazole (MET), ceftriaxone (CEF), and hydroxymetronidazole (MET-OH) in plasma, and CEF in ultra-filtrate (CEF ^uf^) at LLOQ, LOQ, MOQ and HOQ.

Intra-assay (n=6)	Compound	Nominal concentration (µg/ mL)	Mean estimated concentration (µg/ mL) ±SD	Precision (CV %)	Accuracy (%)
	MET	0.05	0.051 ± 2.0	3.9	101.9
		0.15	0.148 ± 7.7	7.8	98.7
		20	20.44 ± 4.0	3.9	102.2
		40	37.75 ± 5.6	5.9	94.4
	CEF	0.4	0.39 ± 2.5	3.2	97.5
		1.2	1.10 ± 1.5	1.7	91.7
		120	112.02 ± 3.5	3.7	93.3
		240	219.51 ± 6.8	7.5	91.5
	MET-OH	0.02	0.018 ± 1.7	2.6	90.0
		0.06	0.057 ± 4.7	4.9	95.0
		12	11.43 ± 2.5	2.7	95.2
		24	24.49 ± 8.6	8.4	102.0
	CEF ^uf^	0.4	0.41 ± 5.5	5.3	100.9
		1.2	1.27 ± 5.5	5.2	105.8
		120	112.32 ± 5.1	5.5	93.6
		240	253.03 ± 8.2	7.8	105.4
**Inter-assay** **(n=18)**	MET	0.05	0.051 ± 1.4	2.7	101.1
		0.15	0.155 ± 5.6	5.4	103.3
		20	20.59 ± 3.3	3.2	103.0
		40	38.79 ± 5.6	5.8	97.0
	CEF	0.4	0.40 ± 2.7	2.9	100.0
		1.2	1.15 ± 3.8	3.9	95.8
		120	114.54 ± 5.1	5.4	95.4
		240	226.75 ± 5.2	5.5	94.5
	MET-OH	0.02	0.019 ± 1.2	2.3	95.0
		0.06	0.059 ± 5.2	5.3	98.3
		12	12.01 ± 4.8	4.8	100.1
		24	24.51 ± 4.7	4.6	102.1
	CEF ^uf^	0.4	0.43 ± 5.8	7.4	107.2
		1.2	1.22 ± 8.3	8.1	101.6
		120	115.32 ± 5.1	5.3	96.1
		240	251.30 ± 6.2	5.9	104.6

### Stability (ST%)

The results of all the stability studies obtained were well within the acceptable limits of accuracy (± 15%) and precision (CV ≤ 15%) ( Table 3).

**Table 3.** Stability (ST%) of metronidazole (MET), ceftriaxone (CEF), hydroxymetronidazole (MET-OH) with the coefficient of variation (CV%) in plasma and CEF in ultra-filtrate (CEF ^uf^) (n=5).

		MET		CEF		MET-OH		CEF ^uf^	
Stability parameters	Spiked conc. (µg/ mL)	0.15	40	1.2	240	0.06	24	1.2	240
Benchtop stability in matrix (room temperature, 8 h)	Mean of stability of samples	0.16	39.32	1.18	230.4	0.059	24.52	1.19	253.9
	CV %	3.8	1.4	4.4	2.5	1.5	3.5	2.4	1.9
	ST %	105.8	98.3	98.1	96.0	99.5	102.2	99.8	105.8
Freeze-thaw stability (3 freeze-thaw cycles at -20°C)	Mean of stability of samples	0.14	37.40	1.15	228.6	0.058	24.18	1.12	221.3
	CV %	3.2	1.8	3.4	2.7	5.1	4.1	4.4	3.1
	ST %	96.1	93.5	95.8	95.3	97.2	100.7	93.0	92.2
Auto-sampler stability (24 h at 18°C)	Mean of stability of samples	0.15	37.50	1.09	228.9	0.062	22.27	1.18	226.1
	CV %	3.3	4.3	5.7	5.4	5.0	5.6	7.1	9.6
	ST %	101.5	93.7	90.6	95.4	103.3	92.8	98.4	94.2
Long-term stability (90 days at -20°C)	Mean of stability of samples	0.14	37.52	1.11	217.4	0.059	22.08	1.13	221.8
	CV %	6.0	4.7	4.2	7.3	1.4	5.6	3.1	5.6
	ST %	95.2	93.8	92.2	90.6	99.1	92.0	94.4	92.4
Sub-stock solution stability (28 days at -20°C)	**Nominal Conc.** **(µg/ mL)**		**50**		**300**		**30**		
	Mean of stability of samples		47.89		288.39		28.63		
	CV %		3.1		2.8		3.4		
	ST %		95.8		96.1		95.4		

***Sub-stock stability.*** All analytes indicated good stability at the storage temperature, 95.4–96.1% of the original concentration was found after storage period of 28 days.

***Freeze and thaw stability.*** Freeze and thaw stability ( Table 3) was consistent with previously reported data by Silva *et al*. ^12^ and Ilomuanya *et al*. ^15^ for MET stability. Ilomuanya *et al*. ^15^ in his freeze/thaw cycle evaluations indicated that after the fourth freeze/thaw cycle the concentrations of MET was < 90%, suggesting that MET is not very stable after three freeze/thaw cycles. This is however the first reported ultra-filtrate stability data for CEF.

***Short term stability or bench-top stability.*** Plasma samples at low and high quality control levels were kept at room temperature for a minimum of eight hours, then processed and analyzed ( Table 3). Some studies have reported stabilities of metronidazole over a longer duration than in this method ^12, 15^. Our choice for the 8 h period was to report an analytically relevant study under which the three drugs can be analyzed. The results indicated that the drugs were stable and therefore the sample processing procedure outlined within this method can be used to process large number of samples without the risk of sample degradation due to room temperature exposure.

Silva *et al*. ^12^ reported the stability of MET over a period of 48h, the mean stability ranging between 93.6% - 100.6%. This stability data was in agreement with what we have reported in this method, however we report the first stability study of MET-OH and CEF in plasma ultra-filtrate.

***24 h stability in the autosampler.*** The results of post processing stability in Table 3 indicated that all the drugs were stable after 24 h in the autosampler and the integrity of data obtained after such re-assay would not be questionable. Ilomuanya *et al*. ^15^ reported the autosampler stability of MET for 72h, however the data reported showed that MET was stable up to 24h and at 72h, the stability was greatly reduced to 40.6%–58.7%.

***Long term stability at -20°C.*** The stability data reported in this study show that all the analytes were stable (90.6%–99.1%) within the period investigated. Since the stability at -20°C was acceptable, there was no need to evaluate the stability at -80°C, as our aim was to report a method that is affordable to resource limited laboratories.

***Carry-over.*** No significant peak indicating carry-over was detected.

***Matrix effect (ME%).*** The protein precipitation method of sample preparation is known to be prone to matrix effect ^36, 37^. Chromatography of analytes or IS, as well as accuracy of the method may be affected by matrix effect, ion suppression or enhancement, due to co-eluting endogenous components. The matrix effect assessment Figure S2A (iv) revealed that only MET showed interference (ion enhancement) at its retention time. The matrix effect encountered with this method ( Table S3) was much lower than in the previously reported method ^15^, this could be attributed to the small sample volumes that were used in our sample processing.

$$ME\%\;=\frac{Response_{POEM}}{Response_{NEAT}}\times \text{100\%}.\;(iii)$$

Response _POEM_ is the average concentration of post extraction spiked matrix and Response _NEAT_ is the average concentration of the analyte in a neat solution.

The samples were prepared at two concentrations and the matrix effect determined as 107.6% (0.15 µg/mL) and 102.1% (40 µg/mL), *n*=6 at both levels. The values obtained at both levels were above 100% indicating the plasma-induced ion enhancement on the analysis of MET and suggesting that the endogenous compounds increased the signal intensity of the analyte in positive ESI mode. The effect of signal enhancement was higher at low concentration level.

### Incurred sample reanalysis (ISR)

Incurred sample reanalysis conducted on 25 samples showed more than 67% had results within the accepted limits (< 20%) of variation. The mean variation of the analytes for the reanalysis were 5.7% (MET), 7.4% (MET-OH) and 7.0% (CEF), therefore, the reported subject sample analyte concentrations can be considered reliable and a true representation of the drug levels at the respective sampling times. Since sample storage was in plasma form, it was not necessary to perform reanalysis on the plasma ultra-filtrate.

### Application of the method to real patient samples

The Optimising Antibiotic Treatment for Sick Malnourished Children (FLACSAM-PK) study was registered (NCT02746276) at ClinicalTrials.gov ^38^.

The validated method was successfully applied to a pharmacokinetic study of CEF, MET, MET-OH and unbound ceftriaxone in hospitalized children with complicated severe acute malnutrition (SAM) following an oral administration of MET and intravenous administration of CEF over the course of 72 hours.

81 hospitalized children with SAM and requiring IV antibiotics according to WHO and national guidelines were recruited (after obtaining ethical approval from the Kenya Medical Research Institute Scientific and Ethics Review Unit, approval number: KEMRI/SERU/CGMR-C023-3161 and informed consent from the parents/guardians) and treated with an oral dose of 7.5mg/Kg MET (Flagyl ^®^oral suspension, 200 mg/5 mL) three times daily and IV injection of 80 mg/kg CEF (Ceftriaxone Rocephin ^®^, 250 mg) once daily 15 min after metronidazole dose. Blood samples (3.0 mL) were collected into Li-heparinized tubes, a pre-dose sample was taken before administering the drugs. Further sampling at 5, 30, 60 min after ceftriaxone dose and 2, 4, and 8 h after metronidazole dose. The sampling plan was such that each patient had only three blood draws after the base-line sample. The blood was centrifuged (3000 rpm; 5 min), plasma separated and stored at -80°C until analysis time.

**Figure 3.**
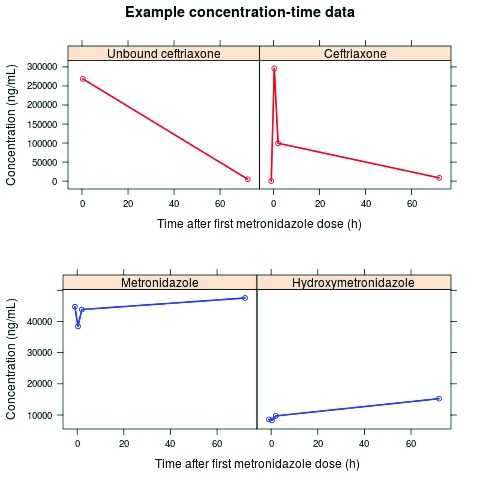
Example concentration-time data of each of the four blood samples (baseline and 3 post first dose), where ceftriaxone, metronidazole and hydroxymetronidazole were quantified. In 2 samples, unbound ceftriaxone was also quantified. This example shows a patient who has clearly taken at least one previous dose of metronidazole prior to study enrolment.

The patient samples were successfully analyzed using this method and no interference of endogenous compounds resulting from altered plasma protein compositions was encountered. Figure 3, shows a concentration–time profile of a baseline and three post-dose samples from a patient who had previously taken at least one metronidazole dose prior to study enrolment, this was evident from the significant levels of metronidazole and hydroxymetronidazole detected from the baseline sample.

We also addressed the recommendations by Wong *et al*. ^17^, as this method allows for direct measurement of unbound ceftriaxone from only 25 µL plasma ultra-filtrate. Figure 4 shows representative chromatograms of processed plasma samples from one of the study participants.

**Figure 4A.**
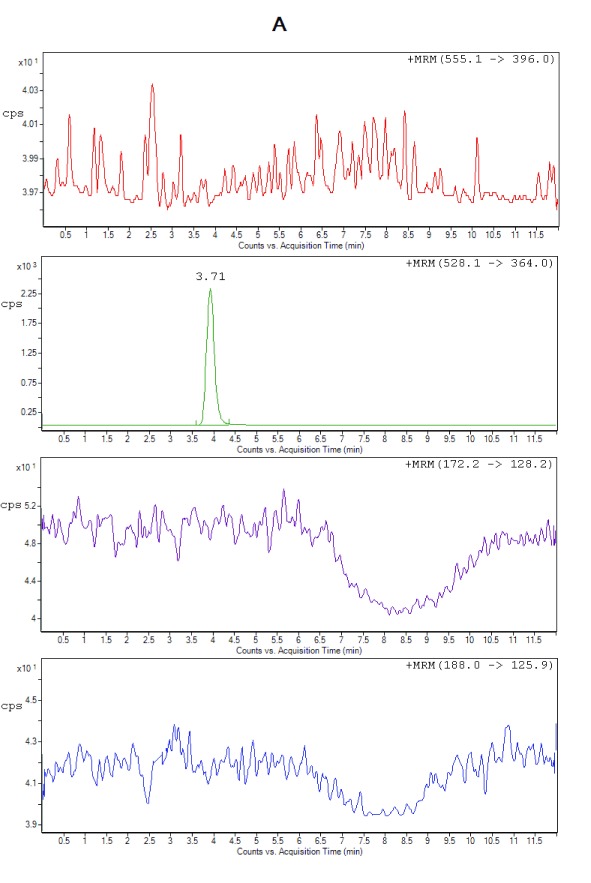
Representative chromatograms from processed plasma study sample at baseline before drug administration with undetectable levels of the drugs.

**Figure 4B.**
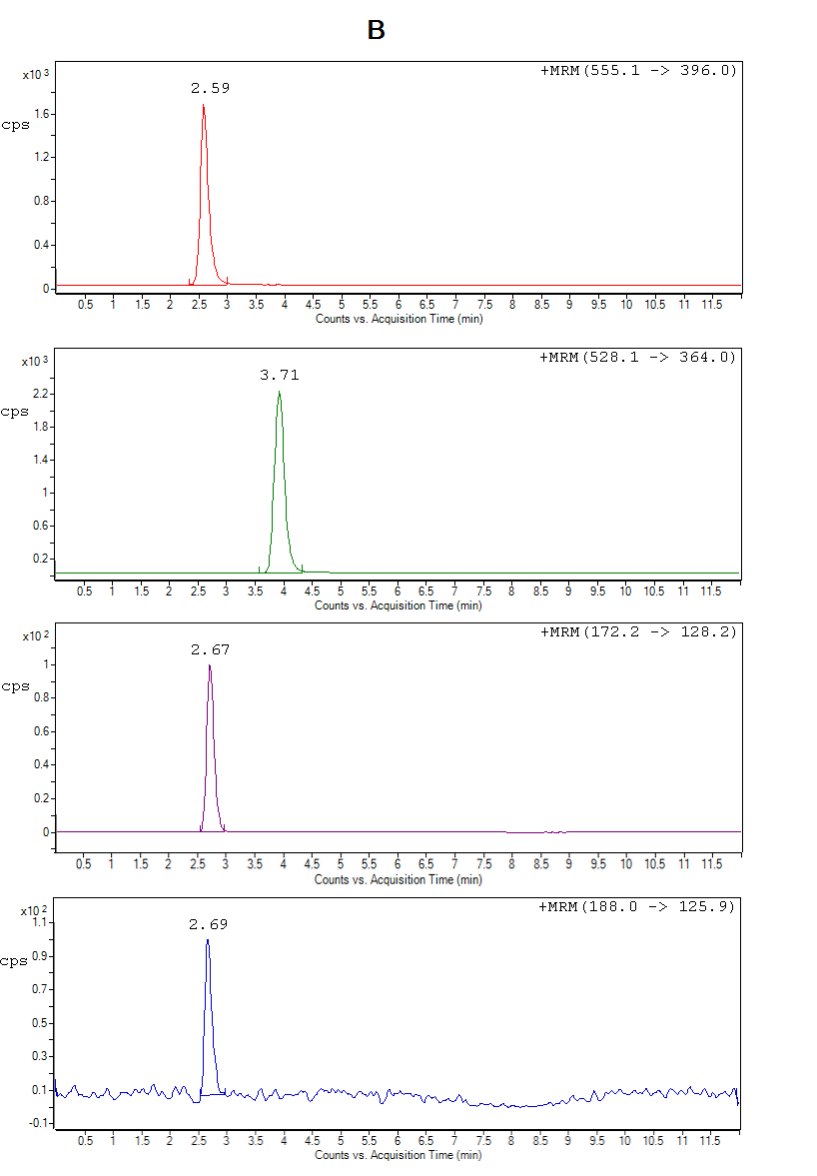
Representative chromatograms of ceftriaxone 266.27µg/mL (RT 2.59 min), metronidazole 2.54µg/mL (RT 2.67 min), hydroxymetronidazole 0.13µg/mL (RT 2.69 min) and cefuroxime (IS) (RT 3.71 min) from processed plasma study sample at 5 min after administering ceftriaxone IV.

**Figure 4C.**
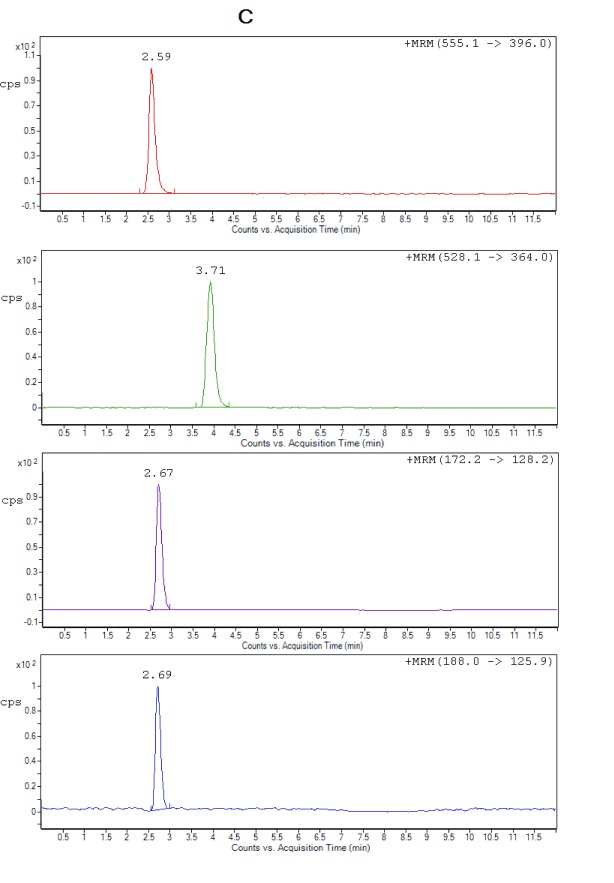
Representative chromatograms of ceftriaxone 74.39µg/mL (RT 2.59 min), metronidazole 1.99µg/mL (RT 2.67 min), hydroxymetronidazole 0.66µg/mL (RT 2.69 min) and cefuroxime (IS) (RT 3.71 min) from processed plasma study sample at 30 min after administering ceftriaxone IV.

## Conclusions

The validated HPLC–ESI–MS/MS method allowed the simultaneous quantitation of metronidazole, hydroximetronidazole, ceftriaxone from only 50 µL human plasma, and of unbound ceftriaxone from 25 µL plasma ultra-filtrate. It provided simple and rapid analyses, as well as sensitive and reliable results. Thus, this method is suitable for routine high-throughput analyses and may be successfully applied to pharmacokinetic and bioequivalence of multiple doses evaluated in the present work in human subjects. The small sample volumes used makes it applicable to pediatric pharmacokinetics and bioequivalence studies, in which large sample volumes maybe unethical or impractical to obtain.

## Supplementary materials

**Figure S1.** MRM product ion spectra of protonated (i) CEF (m/z 555.1→m/z 396.0), (ii) MET (m/z 172.2→m/z 128.2), (iii) MET-OH (m/z 188.0→m/z 125.9) and ammonium adduct of (iv) CEFU (m/z 528.1→m/z 364.0).

**Figure S2.** Representative chromatograms of a direct post column infusion of blank extracted plasma (A, i-iv), MET at ULOQ (A, v) and a blank extracted neat solution showing absence of matrix effect (B, i-iv).

**Table S1.** Regression parameters for ceftriaxone (CEF), metronidazole (MET) and hydroxymetronidazole (MET-OH) in spiked plasma.

**Table S2.** Extraction recoveries of ceftriaxone (CEF), metronidazole (MET), hydroxymetronidazole (MET-OH) and cefuroxime (CEFU) from spiked plasma samples and in ultra-filtrate. Standard deviation (SD); coefficient of variation (CV); internal standard (IS); n=6.

**Table S3.** Matrix effects (ME %) for metronidazole (MET) in 6 plasmas. Standard deviation (SD); coefficient of variation (CV); internal standard (IS); n=6.
