# Supplementary material for: Liquid chromatography–tandem mass spectrometry for the simultaneous quantitation of ceftriaxone, metronidazole and hydroxymetronidazole in plasma from seriously ill, severely malnourished children
Source: Wellcome Open Res. 2018 Jan 30;2:43. Originally published 2017 Jun 19. [Version 2] doi: 10.12688/wellcomeopenres.11728.2 (PMC5801568; doi:10.12688/wellcomeopenres.11728.2)
Supplement: Supplementary file 1 [file wellcomeopenres-2-14807-s0000.tgz › 49c8de14-0a2e-4ebf-adbd-c39407c6c8aa.pdf]

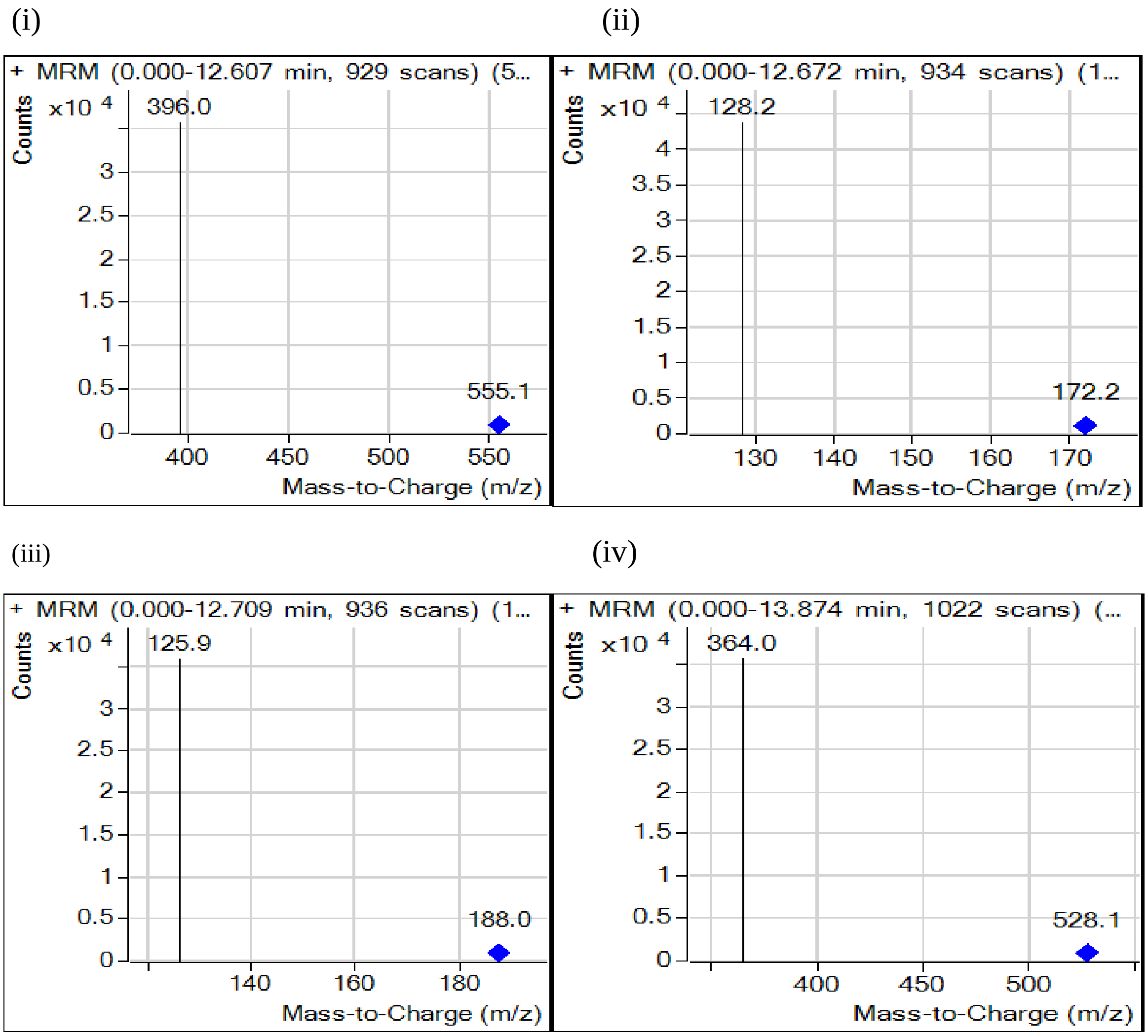

Figure S1. MRM product ion spectra of protonated (i) CEF (*m/z* 555.1→*m/z* 396.0), (ii) MET (*m/z* 172.2→*m/z* 128.2), (iii) MET-OH (*m/z* 188.0→*m/z* 125.9) and ammonium adduct of (iv) CEFU (*m/z* 528.1→*m/z* 364.0).
