# Supplementary material for: Liquid chromatography–tandem mass spectrometry for the simultaneous quantitation of ceftriaxone, metronidazole and hydroxymetronidazole in plasma from seriously ill, severely malnourished children
Source: Wellcome Open Res. 2018 Jan 30;2:43. Originally published 2017 Jun 19. [Version 2] doi: 10.12688/wellcomeopenres.11728.2 (PMC5801568; doi:10.12688/wellcomeopenres.11728.2)
Supplement: Supplementary file 2 [file wellcomeopenres-2-14807-s0001.tgz › 109c6595-462d-4bcb-8c94-7fc55867aa66.pdf]

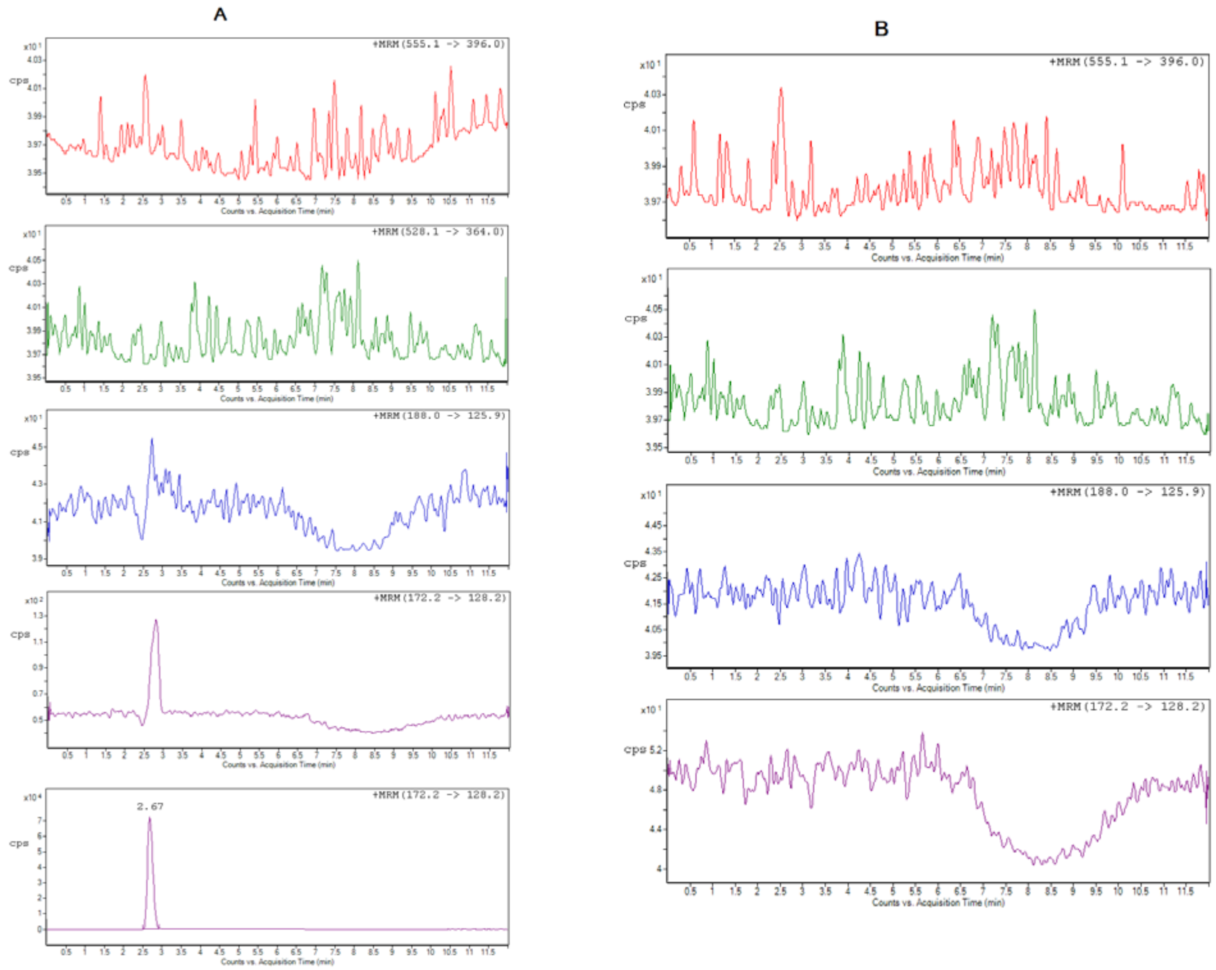

Figure S2. Representative chromatograms of a direct post column infusion of blank extracted plasma (A, i-iv), MET at ULOQ (A, v) and a blank extracted neat solution showing absence of matrix effect (B, i-iv)
