# Supplementary material for: Liquid chromatography–tandem mass spectrometry for the simultaneous quantitation of ceftriaxone, metronidazole and hydroxymetronidazole in plasma from seriously ill, severely malnourished children
Source: Wellcome Open Res. 2018 Jan 30;2:43. Originally published 2017 Jun 19. [Version 2] doi: 10.12688/wellcomeopenres.11728.2 (PMC5801568; doi:10.12688/wellcomeopenres.11728.2)
Supplement: Supplementary file 3 [file wellcomeopenres-2-14807-s0002.tgz › ea95b5ad-0c47-4798-9f2b-0c3957b71235.pdf]

Table S1. Regression parameters for ceftriaxone (CEF), metronidazole (MET) and hydroxymetronidazole (MET-OH) in spiked plasma

**Compound**	**Linearity regression equation**	**Correlation coefficient (r^2± SD)** ***n=6***	**Linear range (μg/ mL)**	**LOD (μg/ mL)**
CEF	y = 3.4E^{-06}x + 0.000351	0.9948± 0.001	0.4- 300	0.005
MET	y = 6.1E^{-04}x + 0.048817	0.9965± 0.003	0.05- 50	0.002
MET-OH	y = 6.8E^{-05}x + 0.002427	0.9968± 0.002	0.02- 30	0.001
CEF^{uf}	y = 6.3E^{-06}x + 0.000588	0.9985± 0.001	0.4- 300	0.005

CEF^{uf}: ceftriaxone in ultra-filtrate
