# Supplementary material for: Liquid chromatography–tandem mass spectrometry for the simultaneous quantitation of ceftriaxone, metronidazole and hydroxymetronidazole in plasma from seriously ill, severely malnourished children
Source: Wellcome Open Res. 2018 Jan 30;2:43. Originally published 2017 Jun 19. [Version 2] doi: 10.12688/wellcomeopenres.11728.2 (PMC5801568; doi:10.12688/wellcomeopenres.11728.2)
Supplement: Supplementary file 4 [file wellcomeopenres-2-14807-s0003.tgz › fe9244d8-2ffb-4606-a8ed-a8eaf5d4c72d.pdf]

Table S2. Extraction recoveries of ceftriaxone (CEF), metronidazole (MET), hydroxymetronidazole (MET-OH) and cefuroxime (CEFU) from spiked plasma samples and in ultra-filtrate. Standard deviation (SD); coefficient of variation (CV); internal standard (IS); n=6

Compound	Spiked concentration (µg/ mL)	Mean recovery ± SD (%)	CV (%)
CEF	1.20	94.5 ± 3.6	3.8
	240	93.4 ± 6.0	6.4
MET	0.15	102.3 ± 6.5	6.3
	40.0	94.0 ± 4.1	4.3
MET-OH	0.06	96.4 ± 5.6	5.9
	24.0	102.7± 5.7	5.6
CEFU (IS)	0.2	93.8± 4.7	5.0
CEF^{uf}	1.20	106.2 ± 5.9	5.6
	240	103.0 ± 5.5	5.4
$CEFU^{uf}$ (IS)	0.2	97.6 ± 1.3	1.2

CEF^{uf}: ceftriaxone in ultra-filtrate
$CEFU^{uf}$: cefuroxime in ultra-filtrate
