# Supplementary material for: Liquid chromatography–tandem mass spectrometry for the simultaneous quantitation of ceftriaxone, metronidazole and hydroxymetronidazole in plasma from seriously ill, severely malnourished children
Source: Wellcome Open Res. 2018 Jan 30;2:43. Originally published 2017 Jun 19. [Version 2] doi: 10.12688/wellcomeopenres.11728.2 (PMC5801568; doi:10.12688/wellcomeopenres.11728.2)
Supplement: Supplementary file 5 [file wellcomeopenres-2-14807-s0004.tgz › 4bd56488-dda0-4d1b-a248-26510cf1111f.pdf]

Table S3. Matrix effects (ME %) for metronidazole (MET) in 6 plasmas. Standard deviation (SD); coefficient of variation (CV); internal standard (IS); n=6

	**Low concentration (0.15 µg/mL)**			**High concentration (40 µg/ mL)**		
	POEM	NEAT	**ME%**	POEM	NEAT	**ME%**
Plasma 1	0.160034	0.141475	113%	42.1406	41.14057	102%
Plasma 2	0.123579	0.141016	88%	40.5073	40.50726	100%
Plasma 3	0.169389	0.147062	115%	40.3619	39.36193	103%
Plasma 4	0.174078	0.138810	125%	42.5051	41.50505	102%
Plasma 5	0.123743	0.143091	86%	41.6815	39.68151	105%
Plasma 6	0.173683	0.147440	118%	40.0669	40.06685	100%
		Mean	107.6%		Mean	102.1%
		SD	16%		SD	2%
		CV	15%		CV	2%
