# Supplementary material for: Liquid chromatography–tandem mass spectrometry for the simultaneous quantitation of ceftriaxone, metronidazole and hydroxymetronidazole in plasma from seriously ill, severely malnourished children
Source: Wellcome Open Res. 2018 Jan 30;2:43. Originally published 2017 Jun 19. [Version 2] doi: 10.12688/wellcomeopenres.11728.2 (PMC5801568; doi:10.12688/wellcomeopenres.11728.2)
Supplement: Peer review history

# Peer review: Referee response for version 2

**DOI:** 10.21956/wellcomeopenres.14807.r30386

**Published:** 2018-02-21

**Authors:** Sandrine Lefeuvre

**Affiliations:** 1 Laboratory of Biochemistry, CHR Orléans, Orléans, France

In the "Preparation of analytical standards", QC levels (LLOQ) are missing.

 The exception of that, I have read this submission. I believe that I have an appropriate level of expertise  to confirm that it is of an acceptable scientific standard.

I have read this submission. I believe that I have an appropriate level of expertise to confirm that it is of an acceptable scientific standard.

# Peer review: Referee response for version 2

**DOI:** 10.21956/wellcomeopenres.14807.r30385

**Published:** 2018-02-06

**Authors:** I-Lin Tsai

**Affiliations:** 1 Department of Biochemistry and Molecular Cell Biology, Taipei Medical University, Taipei, Taiwan

The revised manuscript can be accepted to be indexed.

I have read this submission. I believe that I have an appropriate level of expertise to confirm that it is of an acceptable scientific standard.

# Peer review: Referee response for version 2

**DOI:** 10.21956/wellcomeopenres.14807.r30387

**Published:** 2018-02-02

**Authors:** Pascal Houzé

**Affiliations:** 1 CNRS (French National Center for Scientific Research) UMR8258 - U1022, Faculty of Pharmacy, Paris Descartes University, Paris, France

In view of the authors' replies to the questions asked in my first report and in view of the changes made to the text and figures to answer these questions, I give a favorable opinion to the publication of this article.

I have read this submission. I believe that I have an appropriate level of expertise to confirm that it is of an acceptable scientific standard.

# Peer review: Referee response for version 1

**DOI:** 10.21956/wellcomeopenres.12670.r26950

**Published:** 2017-11-17

**Authors:** Pascal Houzé

**Affiliations:** 1 CNRS (French National Center for Scientific Research) UMR8258 - U1022, Faculty of Pharmacy, Paris Descartes University, Paris, France

The authors report the development of the determination of ceftriazone and metronidazole in children by liquid chromatography coupled with mass spectrometry. Therapeutic adaptation in children is particularly important and especially in malnourished children. The theme developed by the authors is therefore totally up to date.

 In general, the article is well constructed and the validation of method correctly performed in part on the study of stability performed under very varied conditions.

 However, different points need to be clarified:

**The introduction:**

- Why did the authors not also measure the acid metabolite of metronidazole which is active as the parent molecule and the alcohol metabolite? To be explained by the authors

**The material and methods**

- Why the authors dilute the eluent to 1/5 in an aqueous solution of 20% methanol. Why such a large dilution? Why use an aqueous solution of methanol to dilute and not use mobile phase A?
- Would it not have been better to evaporate acetonitrile and take up the dry residue with mobile phase?
- For the quantification of the unbound fraction why do the authors start with 300 μl of serum to finally dilute in 1 ml of aqueous solution of methanol. Why not use less plasma and not dilute in such a large volume?
- The 300 μL used are a little high to qualify the method of micromethode as done by the authors
- For the quantification of the unbound fraction, what is the interest of incubating the plasma for 1 hour at 37 ° C before proceeding to ultrafiltration? Authors should explain this step
- the linearity domains for each molecule must be indicated in the paragraph corresponding to the preparation of the analytical standards
- how are the controls prepared for the study of the precision and accuracy of the method

**The results and discussion**

- In the section selectivity I do not understand the legend of Figure 2. In the text the authors speak of 4 chromatograms: Extracted blank, blank plasma spiked with IS, a spiked plasma with the analytes at LLOQ and ULOQ. Figure 2 shows only 3 chromatograms (A, B and C) and to my avsi, the legends indicated do not correspond to the chromatograms presented. This point is major and must be clarified by the authors
- How did the authors determine the ULOQ values ​​for all the measured compounds?
- For me the authors chose a bad example to illustrate their method of dosage. They should choose another child for whom there is no metronidazole at time T0. On the other hand, the chromatograms presented in FIG. 4 should correspond to those of the kinetics presented. Indeed, in FIG. 4A, chromatogram before injection, there is no peak of metronidazole nor of its metabolite, so this does not correspond to the chromatograms of the kinetics of FIG. 3
- Why was the determination of the unbound fraction of ceftriazone made only at 2 times and the determination of the total?
- How can the authors explain a very high concentration of free ceftriazone at T0 while the total form is undetectable at the same time? form at 4 times?

In conclusion, subject to making the changes mentioned above and especially to review the clinical illustration part of the end of article, this manuscript could be accepted for indexing.

I have read this submission. I believe that I have an appropriate level of expertise to confirm that it is of an acceptable scientific standard, however I have significant reservations, as outlined above.

# Response

**Published:** 2017-12-10

**Authors:** Karin Kipper

**Affiliations:** St George's, University of London, UK

**Responses to the comments by Pascal Houzé.**

 The authors report the development of the determination of ceftriazone and metronidazole in children by liquid chromatography coupled with mass spectrometry. Therapeutic adaptation in children is particularly important and especially in malnourished children. The theme developed by the authors is therefore totally up to date.

 In general, the article is well constructed and the validation of method correctly performed in part on the study of stability performed under very varied conditions.

 However, different points need to be clarified:

 The introduction:

- Why did the authors not also measure the acid metabolite of metronidazole which is active as the parent molecule and the alcohol metabolite? To be explained by the authors

**Response: Respectfully, it is true that both metabolites are pharmacologically active. The major active metabolite however is the alcohol metabolite, we therefore measured the metabolite that would cause a significant clinical effect on the patient. (Manuscript changed to explain this).**

 The material and methods

- Why the authors dilute the eluent to 1/5 in an aqueous solution of 20% methanol. Why such a large dilution? Why use an aqueous solution of methanol to dilute and not use mobile phase A?

**Response:**

**This study involved analysis of samples from multiple dosing with the study drug, high concentrations thus necessitated the large dilutions to levels optimal for MS detection.**

**The method aimed at quantifying multiple drugs with varied physicochemical properties simultaneously. Whereas use of mobile phase A looks conventional, it was tried and found unsuitable due to poor chromatographic peak shapes.**

 Would it not have been better to evaporate acetonitrile and take up the dry residue with mobile phase?

**Response:**

**Respectfully, whereas evaporating acetonitrile and reconstituting the residue could have resulted into better sample purification, we were not keen to adopt that method since we found it much laborious and time consuming. Furthermore the drug levels we anticipated were high enough and pre-concentration was not our priority.**

 For the quantification of the unbound fraction why do the authors start with 300 μl of serum to finally dilute in 1 ml of aqueous solution of methanol? Why not use less plasma and not dilute in such a large volume?

**Response:**

**The ultrafiltration device used to obtain the ultrafiltrate by filtration retain plasma proteins and only the ultrafiltrate to pass through. If smaller volumes of plasma were used, not sufficient ultrafiltrate would be obtained with the recommended maximum speed of x 2000g.**

 The 300 μL used are a little high to qualify the method of micromethode as done by the authors

**Response:The qualification is relative to the previous work done in other publications where sample volumes as high as 500mL were used to determine free fractions using a similar technique of ultrafiltration.**

 For the quantification of the unbound fraction, what is the interest of incubating the plasma for 1 hour at 37 ° C before proceeding to ultrafiltration? Authors should explain this step

**Response:**

**Kindly, this is well explained in the 1 ^st^ paragraph of results and discussion.**

**The equilibrium between the bound and free fractions of ceftraiaxone is temperature dependant, maintaining a physiological temperature through the process is key for a more realistic representation of unbound fractions of the drug.**

 the linearity domains for each molecule must be indicated in the paragraph corresponding to the preparation of the analytical standards

**Response:**

**Manuscript changed to include the linearity domains for each molecule under preparation of analytical standards.**

 how are the controls prepared for the study of the precision and accuracy of the method

**Response: Manuscript changed to include the information.**

 The results and discussion

 In the section selectivity I do not understand the legend of Figure 2. In the text the authors speak of 4 chromatograms: Extracted blank, blank plasma spiked with IS, a spiked plasma with the analytes at LLOQ and ULOQ. Figure 2 shows only 3 chromatograms (A, B and C) and to my avsi, the legends indicated do not correspond to the chromatograms presented. This point is major and must be clarified by the authors

**Response:**

**This was a mislabeled figure legend. **

- **Figure 2A: Representative chromatograms from extracted zero sample (with IS only), cefuroxime (RT 3.71 min) .Changes made to manuscript.**
- **Figure 2B: Representative chromatograms of ceftriaxone (RT 2.59 min), metronidazole (RT 2.67 min), hydroxymetronidazole (RT 2.69 min) and cefuroxime (IS) (RT 3.71 min) from extracted spiked plasma at LLOQ .Changes made to manuscript.**
- **Figure 2C: Representative chromatograms of ceftriaxone (RT 2.59 min), metronidazole (RT 2.67 min), hydroxymetronidazole (RT 2.69 min) and cefuroxime (IS) (RT 3.71 min) from extracted spiked plasma at ULOQ. Changes made to manuscript.**

How did the authors determine the ULOQ values for all the measured compounds?

**Response: **

- **The ULOQ values were determined from anticipated peak concentrations ranges of the analytes, and ensuring that the calibration points met the accuracy and reproducibility criteria of method validation. (Manuscript changed to include this in the calibration curves and limits of quantification section).**

 For me the authors chose a bad example to illustrate their method of dosage. They should choose another child for whom there is no metronidazole at time T0. On the other hand, the chromatograms presented in FIG. 4 should correspond to those of the kinetics presented. Indeed, in FIG. 4A, chromatogram before injection, there is no peak of metronidazole nor of its metabolite, so this does not correspond to the chromatograms of the kinetics of FIG. 3

**Response:**

**Manuscript changed for Fig.4 to correspond to the concentration-time data of kinetics in Fig.3**

 Why was the determination of the unbound fraction of ceftriazone made only at 2 times and the determination of the total?

**Response:**

**Unbound ceftriaxone was measured at only 2 time points, the first time point was drawn 5 min after ceftriaxone iv administration (peak). The second time point was taken at the trough (90 min) after drug administration. Protein binding of ceftriaxone is inversely proportional to plasma concentrations, determining unbound ceftriaxone at the two time points still gave sufficient data to characterize protein binding of the drug.**

 How can the authors explain a very high concentration of free ceftriazone at T0 while the total form is undetectable at the same time? form at 4 times?

**Response**:

**Free ceftriaxone was measured at only 2 time points, the first time point was drawn 5 min after ceftriaxone iv administration (not T0). The second time point was taken at the trough (90 min) after drug administration. Baseline sample was not analyzed for free ceftriaxone since no drug was anticipated at this point.**

**The first time point for total ceftriaxone is a baseline sample and thus undetectable level is expected. The 1 ^st^ time point for total ceftriaxone therefore corresponds to the 2 ^nd^ time point for unbound ceftriaxone. (Manuscript edited to clarify this)**

 In conclusion, subject to making the changes mentioned above and especially to review the clinical illustration part of the end of article, this manuscript could be accepted for indexing.

**Response: Thank you.**

# Peer review: Referee response for version 1

**DOI:** 10.21956/wellcomeopenres.12670.r26951

**Published:** 2017-11-16

**Authors:** Sandrine Lefeuvre

**Affiliations:** 1 Laboratory of Biochemistry, CHR Orléans, Orléans, France

- Calibration and QCs preparation was not clearly explained. A paragraph detailing the preparation of calibrators and QCs in plasma and those used for unbound fraction is missing. How many points? Which matrix is used to quantify the free fraction?
- Target antibiotic concentrations should be determined for each patient, depending on the strain and the MIC. Has the MIC been taken into account to build the calibration range?
- Did the authors consider the impact of adding more methanol in preparing the high QC compared to the low QC? Furthermore, I am concerned that the different sample types (i.e. calibrators, QCs, and patient samples) were handled distinctly, especially with respect to the amount of methanol added to the sample prior to extraction.
- Why 6 min for the re-equilibration step of the analytical column? Could you explain?
- Dilution integrity was not experimented to validate the dilution test to be carried out on drug concentration beyond the calibration interval. Considering the wide range of concentrations expected at different stages of a treatment, the dilution process must be validated according to EMA guidelines
- Incurred sample reanalysis is missing. Differences for instance in protein binding, sample inhomogeneity or concomitant medications, may affect the accuracy and precision of the analyte in such samples during processing and storage. It is therefore recommended to evaluate accuracy of incurred samples by reanalysis of study samples. In accordance with FDA and EMA guidelines.
- The 3 paragraphs (below) p9 should be in the Materials and Methods section. Not in the Results section
  p9:   “Accuracy and precision. To evaluate the inter-assay precision and accuracy, six replicates of quality control plasma samples were analyzed together with one independent calibration ….. A similar assessment was done for plasma ultra-filtrate to determine the accuracy and precision for the unbound ceftriaxone.”  
  p9:  Carry-over.   “A processed blank sample was injected after a high concentration calibration standard at the upper limit of quantification (ULOQ).”   
  p9  Matrix effect (ME%). …..”Two different methods were used to access and determine matrix effect. In the first method, regions of ion suppression or enhancement were evaluated by direct post column infusion of a mixture of analytes and IS at high concentration at the rate of 10 μL/min, while injecting a blank extracted plasma.” … And …….“ In the second method, matrix effect (ion enhancement) was evaluated for MET in six different lots of plasma by comparing the response of post extracted plasma samples spiked with 0.15 µg/mL (LLOQ) and 40 µg/mL (ULOQ) of metronidazole with the response of neat standard solutions spiked at similar concentrations.”  
- CEF is highly bound to proteins ; average protein binding of 89.5%.  Could you explain Figure 3? Protein binding seem strongly affected.

I have read this submission. I believe that I have an appropriate level of expertise to confirm that it is of an acceptable scientific standard, however I have significant reservations, as outlined above.

# Response

**Published:** 2017-12-10

**Authors:** Karin Kipper

**Affiliations:** St George's, University of London, UK

**Responses to the comments by Sandrine Lefeuvre.**

 Calibration and QCs preparation was not clearly explained. A paragraph detailing the preparation of calibrators and QCs in plasma and those used for unbound fraction is missing. How many points? Which matrix is used to quantify the free fraction?

**Response:**

**The manuscript edited to include a detailed preparation of calibrators and QC samples in plasma as well as in measurement of unbound fraction.** **Text added to the manuscript:**

**The matrix used to quantify free fraction of ceftriaxone was plasma ultrafiltrate obtained by ultrafiltration of drug-free plasma.**

 Target antibiotic concentrations should be determined for each patient, depending on the strain and the MIC. Has the MIC been taken into account to build the calibration range?

**Response:**

**Yes, this was considered. An extensive discussion of the same is contained in a separate publication on “Dosing of ceftriaxone and metronidazole in infants with severe acute malnutrition”.**

 Did the authors consider the impact of adding more methanol in preparing the high QC compared to the low QC? Furthermore, I am concerned that the different sample types (i.e. calibrators, QCs, and patient samples) were handled distinctly, especially with respect to the amount of methanol added to the sample prior to extraction.

**Response:** **This was considered. Whereas 400μl of 20% methanol was used in both QCs and patient samples during processing, the calibrators and QCs were pre-spiked with only 25 μl (for 425 μl drug-free plasma).**

 Why 6 min for the re-equilibration step of the analytical column? Could you explain?

**Response:** **The gradient elution used resulted in variation in column pressure with change in mobile phase proportions. The 6 min was to allow column pressure to equilibrate to avoid shifting of peaks**.

 Dilution integrity was not experimented to validate the dilution test to be carried out on drug concentration beyond the calibration interval. Considering the wide range of concentrations expected at different stages of a treatment, the dilution process must be validated according to EMA guidelines

**Response: None of the study samples were above upper limit of quantification nor needed dilution. Dilution integrity was not assessed for this method.**

 Incurred sample reanalysis is missing. Differences for instance in protein binding, sample inhomogeneity or concomitant medications, may affect the accuracy and precision of the analyte in such samples during processing and storage. It is therefore recommended to evaluate accuracy of incurred samples by reanalysis of study samples. In accordance with FDA and EMA guidelines.

**Response: Incurred sample reanalysis was done for study samples and mean variation (accuracy of the re-analysis) for all the analytes was 5.7%-7.4%. Information was added to the manuscript.**

 The 3 paragraphs (below) p9 should be in the Materials and Methods section. Not in the Results section

 p9:   “Accuracy and precision. To evaluate the inter-assay precision and accuracy, six replicates of quality control plasma samples were analyzed together with one independent calibration ….. A similar assessment was done for plasma ultra-filtrate to determine the accuracy and precision for the unbound ceftriaxone.”  

 p9:  Carry-over.   “A processed blank sample was injected after a high concentration calibration standard at the upper limit of quantification (ULOQ).”   

 p9  Matrix effect (ME%). …..”Two different methods were used to access and determine matrix effect. In the first method, regions of ion suppression or enhancement were evaluated by direct post column infusion of a mixture of analytes and IS at high concentration at the rate of 10 μL/min, while injecting a blank extracted plasma.” … And …….“ In the second method, matrix effect (ion enhancement) was evaluated for MET in six different lots of plasma by comparing the response of post extracted plasma samples spiked with 0.15 µg/mL (LLOQ) and 40 µg/mL (ULOQ) of metronidazole with the response of neat standard solutions spiked at similar concentrations.”  

**Response: Thank you.** **The manuscript edited to include procedures of accuracy and precision, carry-over and matrix-effect in the methods section.**

 CEF is highly bound to proteins; average protein binding of 89.5%.  Could you explain Figure 3? Protein binding seem strongly affected.

**Response:** **Respectfully, whereas CEF is known to be highly protein bound, inter patient variability is expected in this category of patients due to their age band and also the prevalence of hypoalbuminemia resulting into altered protein binding. We have explained this in a separate publication.**

# Peer review: Referee response for version 1

**DOI:** 10.21956/wellcomeopenres.12670.r26783

**Published:** 2017-10-24

**Authors:** I-Lin Tsai

**Affiliations:** 1 Department of Biochemistry and Molecular Cell Biology, Taipei Medical University, Taipei, Taiwan

In the manuscript entitled “Liquid chromatography–tandem mass spectrometry for the simultaneous quantitation of ceftriaxone, metronidazole and hydroxymetronidazole in plasma from seriously ill, severely malnourished children”, the authors developed and validated a LC-MS/MS method to quantify drugs from plasma. The following are some comments for the manuscript:

1. Are the chromatograms in 2A and 2B generated from blank samples spiked with drugs at LLOQ and ULOQ respectively? Please indicate the peaks in 2A. What are “zero sample LLOQ” and “zero sample ULOQ” in the figure legend? What are the criteria of LLOQ for each analyte? How the authors defined the LLOQ?
2. Please indicate the spiked concentrations in figure 2C?
3. Please indicate the quantified concentrations in Figure 4.
4. Please use true scale (intensity) instead of 100 % for all the figures.

I have read this submission. I believe that I have an appropriate level of expertise to confirm that it is of an acceptable scientific standard, however I have significant reservations, as outlined above.

# Response

**Published:** 2017-12-10

**Authors:** Karin Kipper

**Affiliations:** St George's, University of London, UK

**Responses to the comments by I-Lin Tsai.**

 1. Are the chromatograms in 2A and 2B generated from blank samples spiked with drugs at LLOQ and ULOQ respectively? Please indicate the peaks in 2A. What are “zero sample LLOQ” and “zero sample ULOQ” in the figure legend? What are the criteria of LLOQ for each analyte? How the authors defined the LLOQ?

**Response: This was a mislabelled figure legend. **

- **Figure 2A: Representative chromatograms from extracted zero sample (with IS only), cefuroxime (RT 3.71 min)** . **Changes made to manuscript.**
- **Figure 2B: Representative chromatograms of ceftriaxone (RT 2.59 min), metronidazole (RT 2.67 min), hydroxymetronidazole (RT 2.69 min) and cefuroxime (IS) (RT 3.71 min) from extracted spiked plasma at LLOQ** . **Changes made to manuscript.**
- **Figure 2C: Representative chromatograms of ceftriaxone (RT 2.59 min), metronidazole (RT 2.67 min), hydroxymetronidazole (RT 2.69 min) and cefuroxime (IS) (RT 3.71 min) from extracted spiked plasma at ULOQ.** **Changes made to manuscript.**
- **The LLOQ is defined under the sub-heading “Calibration curves and limits of quantification” as the lowest standard on the calibration curve that the method is able to identify and quantify discretely with a precision** **≤ 20% and with an accuracy within 80%-120%. No change made to manuscript.**

2. Please indicate the spiked concentrations in figure 2C?

**Response: The response in 1 above regarding Fig 2C addresses this.**

 3. Please indicate the quantified concentrations in Figure 4.

**Response: Manuscript edited with additional information to include concentrations at the sampling points as:**

- **Figure 4A: Representative chromatograms from processed plasma study sample at baseline before drug administration with undetectable levels of the drugs.**
- **Figure 4B: Representative chromatograms of ceftriaxone 266.27** **µg/mL (RT 2.59 min), metronidazole 2.54µg/mL (RT 2.67 min), hydroxymetronidazole 0.13** **µg/mL (RT 2.69 min) and cefuroxime (IS) (RT 3.71 min) from processed plasma study sample at 5 min after administering ceftriaxone iv.**
- **Figure 4C: Representative chromatograms of ceftriaxone 74.39µg/mL (RT 2.59 min), metronidazole 1.99µg/mL (RT 2.67 min), hydroxymetronidazole 0.66µg/mL (RT 2.69 min) and cefuroxime (IS) (RT 3.71 min) from processed plasma study sample at 30 min after administering ceftriaxone iv.**

4. Please use true scale (intensity) instead of 100 % for all the figures.

**Response: Figures have been changed as requested.**

# Peer review: Referee response for version 1

**DOI:** 10.21956/wellcomeopenres.12670.r24432

**Published:** 2017-07-24

**Authors:** Laurens Manning

**Affiliations:** 1 Department of Infectious Diseases, Fiona Stanley Hospital, Murdoch, WA, Australia

This is a straightforward methods paper for a simultaneous LCMS assay for ceftriaxone + metronidazole (+metabolite) from malnourished children. The necessity for good quality drug assays for use in vulnerable populations is a critical component for optimized PK, PK/PD and efficacy studies in the future. However, I whilst I agree with the need for such an assay, I have a few concerns about how the data are reported and some of the analytical processes.

1. The assay is being reported in the conclusion as a low volume assay, but 300µL is required for the ultrafiltration component; I am not sure this can really be considered to be a microsampling technique. The total blood volume taken from very young, anaemic, malnourished children should be a consideration in assay development, and it will almost certainly be an issue for ethics review boards.
2. Stability of the plasma assay has been reported. One of the challenges of working in tropical countries is ensuring that the assay is fit for purpose with respect to sample handling in the field. Often there are delays to plasma separation and the samples may have other delays before being placed into freezer conditions. Our group believes that in tropical and resource poor settings, the assay should account for stability at room temp (not just benchtop stability), tropical ambient temperatures and at 4 degrees.
3. As this assay has been reported as a simultaneous assay, it would be good to see the chromatograms overlaid with the 3 analytes and IS.
4. Could the authors please clarify throughout the manuscript whether MRM or SRM has been used?
5. My major critique is that of the example child used for the validation paper; the authors describe how the assay was used successfully for 81 children; but present a time concentration curve from a single child. The T0 samples in this child had >40000ng/mL. The explanation given is that the child must have had prior exposure to metronidazole. Whilst this is likely to be true, I don’t believe this is appropriate for a methods paper that describes the utility of the assay. I would recommend reporting another ‘sample child’ with a clear undetectable result at T0.

I have read this submission. I believe that I have an appropriate level of expertise to confirm that it is of an acceptable scientific standard, however I have significant reservations, as outlined above.

# Response

**Published:** 2017-08-18

**Authors:** Karin Kipper

**Affiliations:** St George's, University of London, UK

**Responses to the comments by** **Laurens Manning.**

 1. The assay is being reported in the conclusion as a low volume assay, but 300µL is required for the ultrafiltration component; I am not sure this can really be considered to be a microsampling technique. The total blood volume taken from very young, anaemic, malnourished children should be a consideration in assay development, and it will almost certainly be an issue for ethics review boards.

**Response: This is a common misconception. For example, a 1ml sample taken at baseline then 3 time points for an 8kg child represents 0.6% of blood volume (80ml/kg) and would not have a measurable impact on haemoglobin concentration. No change to the manuscript.**

 2. Stability of the plasma assay has been reported. One of the challenges of working in tropical countries is ensuring that the assay is fit for purpose with respect to sample handling in the field. Often there are delays to plasma separation and the samples may have other delays before being placed into freezer conditions. Our group believes that in tropical and resource poor settings, the assay should account for stability at room temp (not just benchtop stability), tropical ambient temperatures and at 4 degrees.

**Response: I don’t think anyone should be doing PK studies if they cannot do the separation soon after the blood was drawn. Our samples were immediately taken to labs with AC in Kilifi & Mombasa and Nairobi. We can confirm that is room temperature remained <28 degrees in all sites, therefore additional stability experiments are not needed to cover the sampling time and temperatures. Comment will be added to the manuscript.**

 3. As this assay has been reported as a simultaneous assay, it would be good to see the chromatograms overlaid with the 3 analytes and IS.

**Response: Respectfully, we thought the chromatograms are best as represented for ease of understanding and interpretation by the reader. Overlaid chromatograms would be necessary with UV detection where overlapping peaks could be troublesome in integration, fortunately that is not the case with MS. No change to manuscript.**

 4. Could the authors please clarify throughout the manuscript whether MRM or SRM has been used?

**Response: MRM was employed as depicted from mass transitions in Table 1 and Figures 2 and 4. SRM was investigated during the early stages of compound optimization. The manuscript will be edited to clarify.**

 5. My major critique is that of the example child used for the validation paper; the authors describe how the assay was used successfully for 81 children; but present a time concentration curve from a single child. The T0 samples in this child had >40000ng/mL. The explanation given is that the child must have had prior exposure to metronidazole. Whilst this is likely to be true, I don’t believe this is appropriate for a methods paper that describes the utility of the assay. I would recommend reporting another ‘sample child’ with a clear undetectable result at T0.

**Response: Respectfully, we acknowledge this as a recommendation rather than a critique since the request is to have additional ‘sample child’ with a clear undetectable result at T0 for comparability purposes and ease of interpretation. Full results of this study are being published separately.**
